# Willingness to work in rural areas and the role of intrinsic versus extrinsic professional motivations - a survey of medical students in Ghana

**DOI:** 10.1186/1472-6920-11-56

**Published:** 2011-08-09

**Authors:** Peter Agyei-Baffour, Shuda R Kotha, Jennifer C Johnson, Mawuli Gyakobo, Kwesi Asabir, Janet Kwansah, Emmanuel Nakua, Mawuli Dzodzomenyo, Rachel C Snow, Margaret E Kruk

**Affiliations:** 1 Department of Community Health, Kwame Nkrumah University of Science and Technology, Kumasi, Ghana; 2 Center for Global Health, University of Michigan, Galleria Building, 1214 S. University Ave, 2nd Floor Suite C, Ann Arbor, Michigan 48104, USA; 3 c/o Office of the Provost, College of Health Sciences, University of Ghana, PO Box KB 52, Legon, Ghana; 4 Ministry of Health, Human Resource for Health Directorate, P.O.Box M44, Accra, Ghana; 5 Ministry of Health, Policy, Planning, Monitoring, and Evaluation Directorate, PO Box M44, Accra, Ghana; 6 School of Public Health, Department of Biological, Environmental and Occupational Health Sciences, PO Box LG 13, University of Ghana, Legon, Ghana; 7 Department of Health Behaviour and Health Education, School of Public Health, University of Michigan, USA; 8 Department of Health Policy and Management, Columbia University Mailman School of Public Health, 600 W. 168th Street, Room 606, New York, NY 10032, USA

**Keywords:** Health Manpower, Motivation, Rural Health Services, Ghana

## Abstract

**Background:**

Retaining health workers in rural areas is challenging for a number of reasons, ranging from personal preferences to difficult work conditions and low remuneration. This paper assesses the influence of intrinsic and extrinsic motivation on willingness to accept postings to deprived areas among medical students in Ghana.

**Methods:**

A computer-based survey involving 302 fourth year medical students was conducted from May-August 2009. Logistic regression was used to assess the association between students' willingness to accept rural postings and their professional motivations, rural exposure and family parental professional and educational status (PPES).

**Results:**

Over 85% of students were born in urban areas and 57% came from affluent backgrounds. Nearly two-thirds of students reported strong intrinsic motivation to study medicine. After controlling for demographic characteristics and rural exposure, motivational factors did not influence willingness to practice in rural areas. High family PPES was consistently associated with lower willingness to work in rural areas.

**Conclusions:**

Although most Ghanaian medical students are motivated to study medicine by the desire to help others, this does not translate into willingness to work in rural areas. Efforts should be made to build on intrinsic motivation during medical training and in designing rural postings, as well as favour lower PPES students for admission.

## Background

The World Health Organization estimates that more than 4 million health workers are needed to fill the health workforce gap globally. This includes 2.4 million physicians, nurses and midwives. Fifty-seven countries are defined as having a critical shortage of health staff; of these, 36 are in sub-Saharan Africa. Of the total world's health work force of 59.2 million, Africa has only 3% of the world's health workers in spite of having 25% of the global burden of disease [1,2]. The shortage of health staff cripples the health delivery system. It is also a threat to provision of essential, life-saving interventions such as childhood immunizations, provision of safe water, safe pregnancy and childbirth services for mothers as well as access to treatment for AIDS, tuberculosis and malaria. Health workers are critical to the global preparedness for and response to threats posed by emerging and epidemic-prone diseases. Different interventions have been tried to address these shortages [3-5].

Retaining health staff in rural areas has proven extremely difficult as young professionals increasingly prefer urban postings and health systems do not reward (and through neglect often penalize) rural service [6, 7. 8]. For example, Wilson and colleagues, 2009, Dovlo, 2003 and Kruk and colleagues, 2010, found that rural exposure, poor working conditions, low job satisfaction, political and ethnic problems, and sometimes, civil strife and poor security in most underserved areas, predispose new graduates to select cities [9-11]. Qualitative research has also shown the importance of health care providers' personal characteristics and value systems, such as religious beliefs and sociopolitical convictions, to their motivation towards rural practice. Emigration of skilled professionals to high-income countries is another barrier to adequate staffing of health facilities. A study in Ghana in 2006 on trainee physicians and nurses revealed that the majority had considered emigrating. More physicians (68%) than nurses (57%) considered emigration. These findings imply that achieving improvements in the health status of people living in low-income countries, and particularly, in rural areas, will be extremely difficult and the attainment of the United Nations Millennium Development Goals 4, 5, and 6 by 2015 [12,13], in Ghana is unlikely.

While previous research has looked at incentives and working conditions to promote uptake of rural posts, few studies have focused on motivation crowding and its effect on willingness to accept postings to rural area. Motivation crowding [14] is the conflict between external factors (extrinsic), such as monetary incentives or punishments, and the underlying desire or willingness to work (intrinsic) in areas needed most. Students may have a mix of extrinsic and intrinsic motivations for studying medicine. Extrinsic factors may either undermine or strengthen intrinsic motivation, led by the belief that medicine has the imperative to help others, as enshrined in the Hippocratic Oath [15-17]. Current monetary incentives, which favour urban practice, may crowd-out the intrinsic desire to give back to society by working in underserved areas [18,19]. This could have debilitating effects on health worker retention in rural areas [20-22]. To tackle the maldistribution of human resources for health (HRH), understanding the factors that crowd-out the intrinsic motivation of medical students and their willingness to accept postings to rural underserved area is integral. This paper analyzes the effect of extrinsic versus intrinsic motivational factors on stated willingness to accept postings to rural underserved areas in Ghana.

## Methods

### Study site

Ghana is located in the west coast of Africa with an estimated population of 23 million [23]. It is mainly an agrarian economy with estimated per capita income of USD 1500. More than two-thirds of the economy is rural, with the cities of Kumasi and Accra having the highest population densities due to brisk economic activities, and relatively strong economic and cultural infrastructure [24]. These attributes make the two main cities destinations for rural-urban migration and a highly heterogeneous socio-cultural context. There are four medical schools in Ghana: the University of Ghana (UG), Kwame Nkrumah University of Science and Technology (KNUST), University for Development Studies (UDS), and University of Cape Coast (UCC).

In Ghana, medical education consists of three years of basic science/para-clinical studies, three years of clinical training at a teaching hospital, and a two-year rotating housemanship. The study was conducted with two public universities in Ghana: University of Ghana (UG) in Accra and Kwame Nkrumah University of Science and Technology (KNUST) in Kumasi. These universities were selected because all the fourth year medical students in the public universities had their clinical training at either UG or KNUST at the time of the study. All 310 fourth year medical students in the country were invited to participate in the study; no sampling was conducted. Fourth-year medical students were selected because they had completed the BSc. Human Biology and had also been exposed to field work, but had not yet made their final decisions about rural or urban practice.

### Data collection

Data collection was preceded by discussions with the heads of medical training institutions, who informed the content of the questionnaire and provided access to the student population. The data collection instruments were developed after seven focus group discussions of 6-8 participants in each group facilitated by trained social scientists were held with third and fifth year medical students at UG and KNUST. The themes for the focus group discussion were motivation, willingness to work in deprived areas, experience in the field, and the influence of background characteristics on willingness to work in deprived areas. The survey instrument, which included semi-structured questions as well as a discrete choice experiment, were then pretested and finalized for the study. The questionnaires were administered electronically with Sawtooth Software SSI Web CAPI [25], in computer laboratories in UG and KNUST.

### Ethical considerations

The study received ethics approval from the Ghana Health Service Ethical Review Committee; the UG Medical School; the KNUST Committee on Human Research, Publications, and Ethics; and the University of Michigan Institutional Review Board. All respondents voluntarily participated after the intent and the design of the study had been explained to them and signing informed consent forms.

### Statistical Analysis

The study used STATA v10.1 for statistical analyses [26]. The main outcome of interest was the willingness to work in a deprived area after graduation. Students were asked to rate how likely they were to work in a deprived area on a scale from 1-4, where 1 represented "I will definitely not work in a deprived area;" 2 "I am unlikely to work in a deprived area;" 3 "I am likely to work in a deprived area;" and 4 "I will definitely work in a deprived area." We collapsed this response set to a dichotomous willing (groups 3 or 4) versus unwilling (groups 1 or 2) to practice in a deprived area. We defined deprived area as "a rural area that is distant from the big cities with few social amenities such as schools, roads, pipe-borne water, etc."--as per Ministry of Health definition.

Predictors of interest included motivation (intrinsic and extrinsic), demographic characteristics, and rural exposure. Students were asked to identify the top three factors that motivated them to study medicine from a list of twelve factors identified as important by the focus group discussions. Five intrinsic motivations included: desire to help others, desire to give back to their home community or country, interest in medicine as a subject matter, inspiration by a role model, and loss of a loved one. Seven extrinsic motivation factors included: income of physicians, job security and lifestyle, social status/prestige, proposed by parents, opportunities to travel and work internationally, ability to use new cutting edge technologies, and research opportunities. Respondents were coded as having "strong" intrinsic or extrinsic motivation if ***two or more ***of their motivational factors were intrinsic or extrinsic. Thus, "strong" intrinsic and extrinsic motivation groups were mutually exclusive.

Demographic factors included: sex, age, ethnicity (Akan vs. non-Akan), partnership status (in a relationship vs. not in a relationship), and parental parental professional and educational status (PPES). The Akan peoples (including the Asante, Fante, Kwahu, Akuapem, Bono and others) are the largest ethnic group, representing approximately half of Ghanaians; we grouped all other smaller ethnic groups (Ga/Dangme, Ewe, Guan, Mole-Dagbani, Grussi, Gruma, and Hausa) together as "non-Akans" for this analysis. High parental professional and educational status (PPES) was defined as having a mother and/or father who is a University-trained professional (e.g. doctor, lawyer, engineer, accountant, technical, etc) and low PPES was defined as neither mother nor father is a University-trained professional. Rural (an area with a population less than 5000) exposure factors included: birth location (urban vs. rural), location of pre-medical studies (urban/vs. rural), having ever lived in rural area (from the age of 5 onwards), exposure to rural service in medical training (for a minimum of 6 months).

Bivariate associations and 95% confidence intervals were estimated using logistic regression. In model 1, the influence of strong intrinsic/extrinsic motivation on willingness to accept postings to rural area were assessed. Socio-demographic factors were added to the regression in Model 2, and rural exposure factors were further added in Model 3.

## Results

### Socio-demographic characteristics

Of the 310 eligible medical students, 307 participated in the survey (99%). Of these, five survey files were corrupted by viruses or lost due to computer malfunction; thus the analysis was conducted with 302 total records. The socio-demographic characteristics of respondents are presented in Table 1. Of the 302 respondents recruited for the study, the majority were male (183 or 60.6%), with a mean age of 22.9 (STD 1.4). Most respondents were born in or around urban areas (264 or 87.4%) and had never lived in rural underserved area (75.8%). In terms of socioeconomic status, 173 (57.3%) students came from high PPES families. About half of the respondents (142 or 47%) were exposed to rural service.

**Table 1:** Demographic characteristics and rural exposure of respondents (N = 302)

Variable	Frequency N = 302	Percentage
Sex		
Male	183	60.6
Female	118	39.1
Rather not say	1	0.3
Age mean (STD)	22.9 (1.40)	
Ethnicity		
Akan	137	45.4
Not Akan^1^	161	53.3
Rather not say	4	1.3
Family PPES^2^		
Low	121	40.1
High	173	57.3
Rather not say	8	2.7
Marital status		
Married or in a relationship	119	39.4
Not in a relationship	176	58.3
Rather not say	7	2.3
Birth Area^3^		
Urban	264	87.4
Rural	33	10.9
Rather not say	5	1.7
Ever lived in rural area^4^		
Yes	72	23.8
No	229	75.8
Rather not say	1	0.3
Exposed to rural service^5^		
Yes	142	47.0
No	99	32.8
Missing/Rather not say	61	20.2

1. Akan includes Asante, Fante, Kwahu, Akuapim, Bono, etc; Non-Akan includes Ga/Dangme, Ewe, Guan, Mole-Dagbani, Grussi, Gruma, and Hausa peoples

2. High Family PPES: Mother and/or father is a University-trained Professional (e.g. doctor, lawyer, engineer, accountant, technical, etc); Low Family PPES: Neither mother nor father is a University-trained Professional

3. Urban area defined as a place with more than 5,000 residents; rural area defined as a place with less than 5,000 residents

4. From age five on

5. Participated in outreach or service in a deprived area during medical studies

### Intrinsic and extrinsic motivation and likelihood of working in underserved area

The intensities of current motivational factors are presented in Table 2. Overall, 158 (55.4%) of students stated that they were likely to or definitely would work in an underserved area. More than 6 in 10 respondents (181 or 63.5%) had strong intrinsic motivation. A higher proportion of respondents who had strong intrinsic motivation indicated willingness to work in a rural area, compared to those with weak intrinsic motivation (χ^2 ^= 6.96, p = 0.008). These results were reversed for those with strong extrinsic motivation (χ^2 ^= 6.12, p = 0.013).

**Table 2:** Intrinsic versus extrinsic motivations to study medicine predict reported likelihood to work in an under-served area, 285 Ghanaian Medical Students

		Reported likelihood to work in an underserved area			
**Top 3 factors that motivated them to study medicine**		**Unlikely**		**Likely**	
	**N = 285^1^**	**N = 127**	**%**	**N = 158**	**%**

Intrinsic Motivation ^2^					
Weak (0 or 1/3 factors)	104	57	54.8	47	45.2
Strong (2 or 3/3 factors)	181	70	38.7	111	61.3
Extrinsic Motivation^3^					
Weak (0 or 1/3 factors)	186	73	39.3	113	60.8
Strong (2 or 3/3 factors)	99	54	54.6	45	45.5

1. Excludes 17 missing values for willingness to work rural and/or current motivation.

2. Intrinsic motivation includes: desire to help others, desire to give back to their home community or country, interest in medicine as a subject matter, inspiration by a role model, and loss of a loved one; Pearson χ^2^(1) = 6.9592, p = 0.008.

3. Extrinsic motivation includes: income of physicians, job security and lifestyle, social status/prestige, proposed by parents, opportunities to travel and work internationally, ability to use new cutting edge technologies, and research opportunities; Pearson χ^2^(1) = 6.1208, p = 0.013.

### Regression analysis of motivations and the willingness to accept postings to rural underserved area after graduation

We present the multivariate logistic regression results for the strength of intrinsic motivation and willingness to work in a rural underserved area after graduation in Table 3. In the unadjusted model (Model 1) there was a significant association between strong intrinsic motivation and willingness to work in rural underserved area (1.92[95% CI 1.18-3.13]). In Model 2, with demographic factors, female gender and high PPES were associated with reduced willingness to practice in a deprived area (0.50[95% CI 0.29-0.88]) and (0.42[95% CI 0.24, 0.71]), respectively, while age was associated with greater willingness to practice in a rural area (1.23[95% CI 1.00-1.52]). Rural exposure factors were not significant when added to the model with intrinsic motivation and demographic factors (Model 3). In the adjusted models 2 and 3, motivation was no longer a significant predictor of willingness to practice in a deprived area.

**Table 3:** Multivariate logistic regression results for strength of intrinsic motivation and willingness to work in a rural underserved area after graduation, Ghanaian medical students

	Model 1		Model 2		Model 3	
	OR	CI	OR	CI	OR	CI
Strong Intrinsic Motivation	1.9	1.2-3.1	1.6	1.0-2.8	1.6	0.9-2.9
						
Demographics						
Female			0.5	0.3-0.9	0.5	0.3-1.0
Age			1.2	1.0-1.5	1.1	0.9-1.4
Akan^1^			0.8	0.5-1.3	0.6	0.3-1.1
High family PPES^2^			0.4	0.2-0.7	0.4	0.2-0.7
Married or in a relationship			0.8	0.5-1.4	0.9	0.5-1.7
						
Rural Exposure						
Born in a rural area^3^					1.4	0.5-4.3
Lived in a rural area^4^					1.4	0.7-3.0
Exposed to rural service^5^					1.5	0.8-2.8
						
N	285		264		209	
Likelihood Ratio χ^2^, p	6.95, p = 0.008		33.48, p < 0.001		31.33, p < 0.001	

1. Akan includes Asante, Fante, Kwahu, Akuapim, Bono, etc; Non-Akan includes Ga/Dangme, Ewe, Guan, Mole-Dagbani, Grussi, Gruma, and Hausa peoples

2. High family PPES: Mother and/or father is a University-trained Professional (e.g. doctor, lawyer, engineer, accountant, technical, etc); Low family PPES: Neither mother nor father is a University-trained Professional

3. Urban area defined as a place with more than 5,000 residents

4. From age five on

5. Participated in outreach or service in a deprived area during medical studies

In Table 4 the influence of strong extrinsic motivation on willingness of students to work in rural underserved area is presented. In Model 1, having a strong extrinsic motivation reduced the odds of being willing to accept a job in an underserved area to (0.54[95% CI 0.33-0.88]). In the model adjusting for demographics, Model 2, female gender and high PPES were associated with reduced willingness to practice in underserved areas (0.50[95% CI 0.28-0.87] and (0.42[95% CI 0.24-0.71]) respectively, while age was associated with greater willingness to practice in a rural area (1.24[95% CI 1.00-1.53]). Rural exposure factors in model 3 did not influence the outcome of willingness to work in rural underserved area. In the adjusted models 2 and 3, motivation was no longer a significant predictor of willingness to practice in a deprived area.

**Table 4:** Multivariate logistic regression results for strength of extrinsic motivation and willingness to work in a rural underserved area after graduation

	Model 1		Model 2		Model 3	
	OR	CI	OR	CI	OR	CI
Strong Extrinsic Motivation	0.5	0.3-0.9	0.6	0.4-1.1	0.6	0.3-1.2
Demographics						
Female			0.5	0.3-0.9	0.5	0.3-1.09
Age			1.2	1.00-1.5	1.1	0.9-1.4
Akan^1^			0.8	0.5-1.4	0.6	0.3-1.1
High family PPES^2^			0.4	0.2-0.7	0.4	0.2-0.8
Married or in a relationship			0.8	0.5-1.4	0.9	0.5-1.7
						
Rural Exposure						
Born in a rural area^3^					1.4	0.5-4.3
Lived in a rural area^4^					1.4	0.7-3.1
Exposed to rural service^5^					1.5	0.8-2.8
						
N	285		264		209	
Likelihood Ratio χ^2^, p	6.11, p = 0.01		33.03, p < 0.001		31.4, p < 0.001	

1. Akan includes Asante, Fante, Kwahu, Akuapim, Bono, etc; Non-Akan includes Ga/Dangme, Ewe, Guan, Mole-Dagbani, Grussi, Gruma, and Hausa peoples

2. High family PPES: Mother and/or father is a University-trained Professional (e.g. doctor, lawyer, engineer, accountant, technical, etc); Low family PPES: Neither mother nor father is a University-trained Professional

3. Urban area defined as a place with more than 5,000 residents

4. From age five on

5. Participated in outreach or service in a deprived area during medical studies

## Discussion

We found that twice as many students reported high intrinsic motivation compared to high extrinsic motivation to study and practice medicine. This may reflect the underlying altruistic motivation for many students entering a profession focused on serving others [12,13]. There may also be an element of social desirability bias in the students' responses as intrinsic motivation may be thought to be more socially acceptable than extrinsic motivation. Nonetheless, we found that high extrinsic motivation was associated with low self-reported likelihood of rural practice and that the converse was true for high intrinsic motivation [2,3]. Interestingly, this association lost statistical significance at the 95% confidence level in models with demographic and rural exposure confounders, whereas socioeconomic status (PPES) retained a highly influential role, as discussed below.

In this study, rural origin did not influence students' willingness to practice in rural areas after controlling for intrinsic/extrinsic motivation and demographic characteristics. This is in contrast with studies which have found rural origin to be an important motivator for rural practice [8,17,23]. Our findings highlight the heterogeneity of trends in motivation dynamics for rural practice and the importance of locally-relevant data for decision making. High PPES, measured using parental education and profession, was consistently associated with lack of willingness to work in rural areas. This is concerning as nearly 6 in 10 medical students in this cohort were from high PPES backgrounds--which is typical for Ghanaian medical schools [7]. These findings suggest that admission policies that favour well-to do applicants may be reducing the pool of students willing to consider rural practice. Female gender was also strongly associated with reduced interest in rural practice for women even after controlling for extrinsic/intrinsic motivation and rural exposure variables. This is consistent with similar studies among health staff which revealed that women are less likely to accept positions in remote areas due to varying family reasons; they would like to live where their husbands jobs are, have difficulties convincing their husbands to follow them to rural areas and want their children to have better education in the urban areas [10,11,22]. The studies further explained that female doctors rarely live in the same village as their assigned post and have higher overall absentee rates in rural practice [19,20]. With increasing representation of female healthcare professionals in many places in sub-Saharan Africa, [10,11], it is likely that the supply of health staff to rural underserved areas will remain a major setback if professional motivations are designed to attract more female students to rural practice. Although our study showed a lower proportion of female medical students in Ghana compared to other areas, they are likely to become a more important cadre in the coming years [11,19]. More research is urgently needed to determine how female healthcare professionals' motivations towards rural practice can be better engaged by policy-makers.

The limitations of this study include the possibility of social desirability bias in responses on motivation and likelihood of rural practice, as noted above. Despite the fact that study participants were assured of anonymity, confidentiality, in responding to the questions, some social desirability bias is likely. For this reason, we selected a measure of high intrinsic and extrinsic motivation for use in the regression models. Research comparing students stated intentions with their actual career choices during internship is urgently needed as few studies on matched follow-ups are available. In addition, most the students participating in the study were young and had not yet tasted the rigors of working in a rural area, which may affect their job preferences. Thus the findings of this study may not be applicable to practicing physicians. Finally, these findings are only generalizable to students in the current medical education system. The findings may be different if selection criteria for medical school admission change.

The major strengths of this study are its high response rate of 99% and that its ability to capture an entire population of young medical students who are one of the targets for addressing the rural-urban health staff recruitment imbalance. Surveying practicing physicians would have missed out those who had migrated.

This study has several implications. First, the majority of students profess high intrinsic motivation for rural service. More research is needed to determine the potency of this motivation source in real-life decision making and how to best engage it via HRH policy. It is possible that emphasizing the community service aspect of medical practice and elevating the status of rural primary care in under-graduate and post-graduate training may help narrow the gap between motivation and eventual career choice in favour of rural areas. In addition, well-supervised and supported rural placements in which students experience the rewards of rural practice may help to persuade students who are largely unfamiliar with rural life. However, the success of these rural rotations is likely to depend heavily on having adequate local infrastructure and mentorship [13,23].

Second, admission criteria may need to be reconsidered in light of the strong relationship between high PPES and lack of interest in rural practice. For example, medical school admission slots might be reserved for qualified students from poorer families. These students may not need to come from rural areas as we found that none of the rural exposure factors were significant after controlling for motivation and demographics.

Third, our results suggest that programmes to promote and support rural practice after graduation may have some success. Our focus groups and discrete choice experiment suggested that students may be willing to commit to short-term placements of 2 years or less in rural areas [9]. The Ministry of Health may want to consider the possibility of short contracts that rotate physicians in and out of difficult to staff rural areas.

## Conclusions

The majority of students still claim high intrinsic motivation and therefore it is important to appeal and build on this in medical school curricula and in designing rural postings. However, extrinsic motivation and, perhaps most importantly, PPES, will likely continue to be an important factor in deciding on job postings. Future research should explore how motivations could be directly supported, how motivations are formed, the influence of contextual factors on motivation among medical students, and motivation crowding among practicing health professionals in rural underserved areas. Qualitative work may be especially informative in this effort. Our research also suggests that increasing efforts to recruit medical students from low PPES backgrounds may be the most effective current pathway to increasing the yield of physicians willing to practice in underserved areas.

## List of Abbreviations

AIDS: Acquired Immune Deficiency Syndrome; BSc.: Bachelor of Science; CHARTER: Collaborative Health Alliance for Reshaping Training, Education and Research; CI: Confidence Interval; HRH:Human Resource for Health; KNUST: Kwame Nkrumah University of Science and Technology; PPES: parental professional and educational status; STD: Standard Deviation; UCC: University of Cape Coast; UG: University of Ghana; USD: United Stated Dollars

## Competing interests

The authors declare that they have no competing interests.

## Authors' contributions

PA-B, SRK, JCJ, EN, MG, KA, MD, JK, RCS, and MEK jointly conceived the study and designed the survey. PA-B, JCJ, MG, EN, and KA carried out data collection, under supervision by MEK. All authors were involved in the interpretation of study findings. PA-B, wrote the first draft of the manuscript. All authors reviewed and critically revised the manuscript for important intellectual content and agreed to submit the manuscript for publication.

## Pre-publication history

The pre-publication history for this paper can be accessed here:

http://www.biomedcentral.com/1472-6920/11/56/prepub
